# The effects of dietary fatty acids on bone, hematopoietic marrow and marrow adipose tissue in a murine model of senile osteoporosis

**DOI:** 10.18632/aging.102299

**Published:** 2019-09-25

**Authors:** Ebrahim Bani Hassan, Mostafa Alderghaffar, Fabien Wauquier, Veronique Coxam, Oddom Demontiero, Sara Vogrin, Yohann Wittrant, Gustavo Duque

**Affiliations:** 1 Australian Institute for Musculoskeletal Science (AIMSS), The University of Melbourne and Western Health, St. Albans, VIC, Australia; 2 Department of Medicine-Western Health, The University of Melbourne, St. Albans, VIC, Australia; 3 Ageing Bone Research Program, Sydney Medical School Nepean, The University of Sydney, Penrith, Australia; 4 Human Nutrition Unit, UMR1019, INRA/Université Clermont Auvergne, Clermont-Ferrand, France

**Keywords:** aging, fish oil, marrow adipose tissue, osteoporosis, SAMP8 mouse

## Abstract

Purpose: Marrow adipose tissue (MAT) expansion and associated lipotoxicity are important drivers of age-related bone loss and hematopoietic bone marrow (HBM) atrophy. Fish oil and borage oil (rich in ω3 fatty acids) can partially prevent aged-related bone loss in SAMP8 mice. However, whether preservation of bone mass in this progeria model is associated with MAT volumes remains unknown.

Results: MAT volume fraction (MAT%) showed a negative association with hematopoietic bone marrow (HBM%;r=-0.836, *p*<0.001) and bone (bone%;r=-0.344, *p*=0.013) volume fractions.

Adjusting for multiple comparisons, bone% was higher and MAT% was lower in Fish oil (FO)-supplemented groups vs. controls (*p*<0.001). HBM% did not differ significantly between the four groups. However, in the group supplemented with FO, HBM comprised higher fractions and MAT constituted lower fractions of total marrow vs. controls (p<0.001).

Conclusion: Feeding FO-enriched diet prevented age-related bone and HBM loss, by reducing MAT expansion. Our results further emphasize on the role(s) of MAT expansion in bone and HBM atrophy.

Methods: SAMP8 mice (n>9 /group) were allocated into 4 categories and fed a control ration, FO-, sunflower oil (SFO)- and borage oil-enriched diets for lifetime. Femurs were scanned using microcomputed tomography (μCT) and bone, MAT, and HBM volumes were determined using an image analysis software.

## INTRODUCTION

Marrow adipose tissue (MAT) accumulation and the associated marrow lipotoxicity has been suggested as a major cause of bone and red marrow volume decline in humans and animals [1–5]. There is evidence that MAT-associated lipotoxicity impairs osteoblast function and survival, driving osteoblast towards a pro-apoptotic/ pro-adipogenic phenotype, potentially resulting in osteoporosis [1–4, 6–8]. MAT accumulation, which occurs at the expense of bone and hematopoietic bone marrow (HBM) [1], is associated with decreased bone formation at the proximity of MAT [9], and declined bone mechanical strength [8].

It has previously been shown that lifelong diets (starting at the young age and continuing till the end of life) supplemented with fish oil (FO) and borage oil (both rich in ω3 [n-3] fatty acids) can partially curtail age-related bone loss in SAMP8 mice (a progeria animal model), that interestingly coincided with decreased levels of inflammatory markers [10]. However, it is unknown whether preservation of bone mass in this progeria model is accompanied by reduced MAT volume.

To study the possible roles of MAT in the reported bone mass preservation associated with the supplementation of fatty acids we measured bone, MAT and HBM in the femoral bones of SAMP8 mice. We hypothesized that higher bone volume seen in feeding fatty acids in the SAMP8 mice is associated with low MAT and high HBM volumes.

## RESULTS

Of a total 53 mice in 4 groups, 10 were controls, 20 received FO, 11 received Sun flower oil (SFO) and 12 received borage oil supplements.

**Figure 1:**
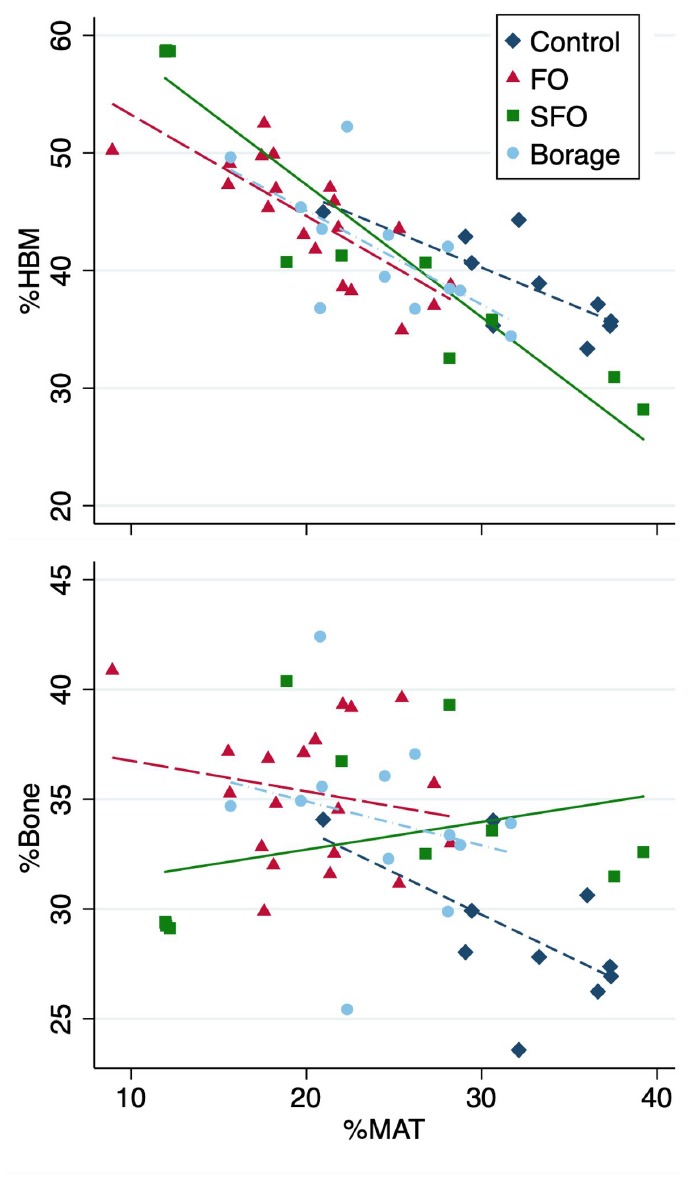
**Associations of total tissue volume percentages of marrow adipose tissue (%MAT) vs hematopoietic bone marrow (%HBM, upper panel) and %bone (lower panel) in the four test diet groups.**

Overall MAT volume (including all groups) showed a strong negative association with HBM (r=-0.836, *p*<0.001), with the strongest association observed within SFO group (r=-0.949; *p*<0.001), followed by FO (r=-0.782; *p*<0.001), control (r=-0.758; *p*=0.011) and borage oil (r=-0.678; *p*=0.015) cohorts (Figure 1). There was an overall negative association between MAT and bone volume (r=-0.344, *p*=0.013), with associations being stronger in the control group (r=-0.586, *p*=0.075). None of the groups receiving fatty acid supplements showed significant associations between bone and MAT volumes (r=-0.225 to 0.332; *p*=0.337 to 0.482 for FO, SFO and borage oil groups respectively; Figure 1).

**Figure 2:**
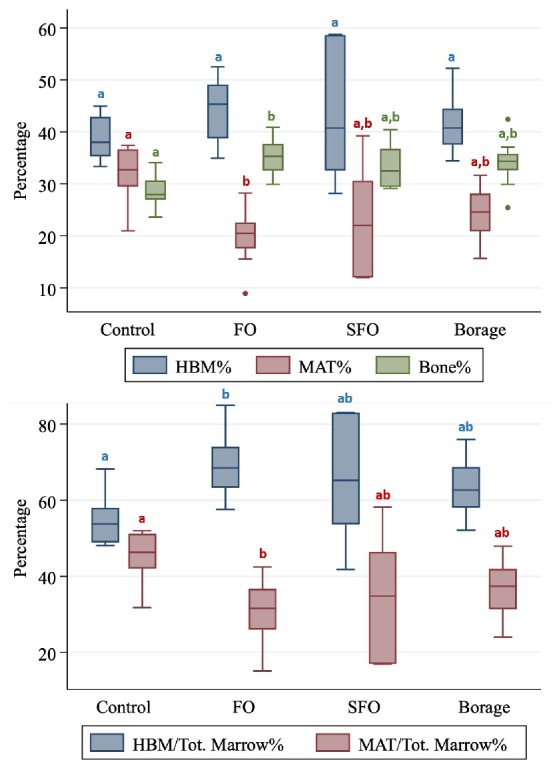
**Hematopoietic bone marrow (HBM%), marrow adipose tissue (MAT%) and Bone (Bone%) volume fractions (Upper panel) and total marrow volume fractions of HBM and MAT compared between four nutritional groups (Control; fish oil [FO], sunflower oil [SFO] and borage oil groups).** Groups with different letter markers are significantly different after adjusting for multiple comparisons (*p*<0.001).

Despite higher bone% and HBM% and lower MAT% values of FO group compared to all other groups, after adjusting for multiple comparisons the differences did not reach significance (Table 1, and Figure 2). In addition, FO group had significantly (*p*<0.001) higher bone volume fraction and lower MAT% compared to controls (Table 1).

**Table 1:** Presented are median and IQR.

**Factor**	**Control (C) (n=10)**	**Fish oil (FO) group (n=20)**	**Sunflower oil (SFO) group (n=11)**	**Borage oil (B) group (n=12)**	**Overall *p*-value**	**Pair-wise comparisons *p*-values**					
						***C vs FO***	***C vs SFO***	***C vs B***	***FO vs SFO***	***FO vs B***	***SFO vs B***
MAT%	32.7 (29.4, 36.6)	20.5 (17.6, 22.6)	22.0 (12.0, 30.6)	24.6 (20.8, 28.1)	<0.001	<0.001	0.041	0.001	0.59	0.035	0.58
HBM%	38.0 (35.3, 42.9)	45.3 (38.8, 49.1)	40.7 (32.5, 58.7)	40.8 (37.6, 44.5)	0.12	NA*	NA*	NA*	NA*	NA*	NA*
Bone%	27.9 (26.9, 30.6)	35.3 (32.5, 37.7)	32.5 (29.4, 36.7)	34.3 (32.6, 35.8)	0.002	<0.001	0.029	0.01	0.074	0.39	0.3
HBM/total marrow%,	53.71 (48.8, 58.0)	68.44 (63.3, 74.0)	65.23 (53.6, 83.0)	62.64 (58.1, 68.7)	0.005	<0.001	0.091	0.01	0.53	0.068	0.71
MAT/total marrow%,	46.29 (42.0, 51.2)	31.56 (26.0, 36.7)	34.77 (17.0, 46.4)	37.36 (31.3, 41.9)	0.005	<0.001	0.091	0.01	0.53	0.068	0.71

*Inter-group comparisons are not presented due to non-significant overall difference. However, it is worth noting that the mean HBM% of fish oil group is at least 12% higher than the other groups.

Due to adjustments for post-hoc pair-wise multiple comparisons (overall p-value), a p<=0.001 is considered statistically significant.

In addition, marrow ratio of HBM was highest and MAT was lowest in the FO group compared to all other groups. After adjusting for multiple comparisons, the differences remained significant against controls (*p*≤0.001; Table 1).

## DISCUSSION

In this study, FO prevented age-related bone and hematopoietic marrow loss by preventing the expansion of MAT. Lifelong supplementation of fatty acids possibly impeded the degree of negative associations of MAT against bone.

The findings of this study confirm the previous reports of the higher bone mineral density of SAMP8 mice fed FO - and borage oil to some degree [10]. However, as the most important finding, our results suggest possible roles that MAT expansion plays in both bone and HBM volume decline in this model; and importantly, the ability of ω3 fatty acids (abundant in FO) to hinder MAT expansion and prevent the associated bone and HBM loss.

The core rationale for selecting 3 types of fatty acid treatment groups was to elucidate whether and how the ω3 vs ω6 fatty acids affect bone and marrow health; and whether the composition of ω3 supplements plays a role. As per Table 2, the most important difference between the effective FO-enriched diet versus less/non-effective other diets was significantly lower ω6/ω3 ratio and presence of 20:5 n−3 fatty acids in fish oil (vs. none in other diets; Table 2).

**Table 2:** The fatty acid composition of the four types of diets used in this study (% of total fatty acids).

**Fatty acid carbon number**	**Standard diet (Harlan 2019)**	**Sunflower oil based diet**	**Borage oil enriched diet**	**Fish oil enriched diet**
12:0	0.71	0.00	0.00	0.00
14:0	0.15	0.05	0.05	0.09
15:0	0.00	0.00	0.00	0.00
16:0	11.35	5.11	6.58	5.03
17:0	0.00	0.02	0.01	0.01
18:0	3.24	2.75	2.89	2.97
19:0	0.00	0.00	0.00	0.00
20:0	0.25	0.19	0.16	0.16
22:0	0.08	0.24	0.12	0.12
**Total SFA**	15.78	8.36	9.81	8.38
16:1	0.15	0.08	0.14	0.19
**18:1 n−9**	**23.4**	**46.89**	**36.84**	**37.77**
18:1 n−7	0.00	0.00	0.21	0.52
20:1	0.54	0.34	1.17	0.73
22:1	0.05	0.00	0.54	0.00
24:1	0.00	0.00	0.34	0.00
Total MUFA	24.14	47.31	39.24	39.21
**18:2 n−6**	**43.40**	**54.23**	**42.04**	**40.51**
**18:3 n−6**	**0.00**	**0.00**	**5.19**	**0.00**
20:4 n−6	0.00	0.00	0.00	0.31
Total PUFA ω-6	54.23	42.04	48.59	40.82
**18:3 n−3**	**5.93**	**2.29**	**2.35**	**2.38**
18:4 n−3	0.01	0.00	0.00	0.52
20:4 n−3	0.00	0.00	0.00	032
**20:5 n−3**	**0.00**	**0.00**	**0.00**	**6.35**
21:5 n−3	0.00	0.00	0.00	0.22
22:5 n−3	0.00	0.00	0.00	0.31
22:6 n−3	0.00	0.00	0.00	1.50
Total PUFA ω-3	**5.94**	**2.29**	**2.35**	**11.60**
Total PUFA	60.16	44.33	50.94	52.42
**LA/ALA**	9.14	18.35	18.46	17.02
**n−6/n−3**	9.13	18.35	20.67	3.52
***Total percent***	100.00	100.00	100.00	100.00

Main lipid differences have been highlighted in bold.

Fat redistribution with aging (from subcutaneous fat into muscle, bone marrow and viscera) [6, 7, 11, 12] leads to a pathological state where adipocytes lose the ability to metabolize triglycerides, respond to hormones (e.g. insulin), and produce abnormal endo-, para- and auto-crine factors (e.g. inflammatory cytokines, leptin, adiponectin and resistin) [6, 7, 12]. Also, palmitic acid is profusely produced by MAT in human cells *in vitro* [2] and *in vivo* [13], which is toxic to osteoblasts [4] and capable of inducing a pro-inflammatory response within marrow [14]. In addition, it has been shown that adipose tissue releases only some or none of the expressed IL-6 and TNFα into the blood stream [15]. The reported lack of correlation between MAT and circulating cytokines [1] indicates high concentrations of such inflammatory mediators in the marrow milieu and auto/paracrine activity. The combined effects of inflammation and aging (inflammaging) on osteoporosis [16] and anemia [17] are well-known, and the above supports the concept that MAT can induce direct paracrine lipotoxicity and inflammation on bone and HBM tissues and drive their atrophy.

Polyunsaturated fatty acids and particularly the ω3 variety have already been linked to bone health in humans and animal models [18, 19]. Interestingly ω3 fatty acids are associated with preventing endoplasmic reticular stress and saturated fatty acid–mediated activation of innate immune processes such as production of interleukin (IL)-1, IL-6 and TNF, and finally bone preservation in ovariectomized mice [19–21]. Decreased osteoclastogenesis [19] and osteoclastic activity [10] has been shown as an effect of ω3 fatty acids supplementation. Considering the ability of inflammation [22, 23] and lipotoxicity (particularly by MAT) [24, 25] in inducing osteoclastogenesis, all evidence point at the potentials of the ω3 fatty acids on blocking adipose tissue-induced inflammation, that preserves bone mass. In fact, fish oil suppresses bone resorption by inhibiting osteoclastogenesis by lowering expression of macrophage colony-stimulating factor (M-CSF), PU.1, microphthalmia-associated transcription factor (MITF), and importantly receptor for activation of NFκB (RANK) and RANK ligand (RANKL) [26].

Furthermore, Omega 6 fatty acids may stimulate the uptake and depot of extracellular fats within cells [27], and ω3 fatty acids may potentially be able to compete them and slow down adipogenesis and associated lipotoxicity and inflammaging in the marrow milieu. In agreement with this hypothesis, feeding mice with a FO-rich diet can decrease PPAR-γ gene expression [10] in favour of osteoblastogenic and against adipogenic commitment of stem cells. Additionally, FO has been shown to reduce MAT volume [28] and promote hematopoiesis in the bone marrow [29]. However, to our knowledge the positive effects of FO on the prevention of MAT expansion and its protective effects on both bone and HBM - which is of high pathophysiological importance for both age-related bone loss and anemia - have not been reported before.

Considering the recent reports of very close direct interdigitated cellular processes and indirect contact between MAT, HBM, osteoblasts and bone lining cells with abundant fat vacuoles in-transit from MAT into these tissues [30], the chances of both inflammaging and lipotoxic effects of MAT on both bone and HBM is very likely.

Our finding that prevention of MAT expansion can lead to higher ratios of both bone and HBM in this progeria model resonates with the findings of Bani Hassan et al. [1], who reported that MAT expansion in older men accompanies both bone and HBM atrophy, which can lead to both osteoporosis and anemia. These findings are of significance, as both anemia and osteoporosis are interrelated age-associated conditions that are of high prevalence in older people, with potentials to cause falls and fractures [31–35]. Blocking MAT expansion by nutrition, medication or a combination of the two approaches, may open new horizons for the prevention and treatment of both anemia and osteoporosis.

Other murine models of progeria (e.g. Lamin A- and Zmpste24-deficient, Wrn^−/−^, Terc^−/−^, PolgA^mut/mut^, Klotho^−/−^, PolG and XPD strains) also do show osteoporosis [36]. Interestingly, PolG and PolgA^mut/mut^ strains also show concomitant anemia and osteoporosis [36], that akin to SAMP8 model, may imply similar MAT lipotoxicity mechanisms inducing both conditions in both models. However, whether all other progeria mice have both conditions or not has not been reported, and should be investigated case by case.

Unlike other reports [19, 37], we did not find evidence on the negative effects of ω6 fatty acids (SFO group) on bone, which agrees with the observation that ω6 fatty acids had no incremental effects on MAT either; that is also in agreement with previous reports of no difference in the bone mineral density of sunflower oil fed mice vs controls [10]. Albeit, the potentials of ω6 fatty acids to affect bone health is complex, and depends on their constituents (e.g. linoleic to linolenic acid ratios) or the ratios of ω3/ω6 [38].

**Figure 3:**
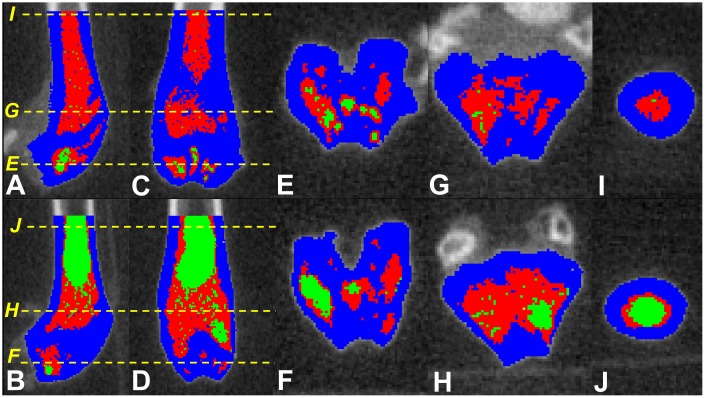
**Screen shots of the tagged images of the distal femora of SAMP8 mice fed fish oil supplemented (upper panel) and control (lower panel) diets.** Parasagittal (**A** and **B**) and coronal/frontal (**C** and **D**) plane sections: (**E**–**J**) *(yellow)* dotted lines represent the cross-sectional planes that images (**E**–**J**) display. Those fed fish oil-enriched diet appear to have higher bone (blue), hematopoietic marrow (red) and lower marrow adipose tissue (green) compared to controls.

Major strengths of this study are the longitudinal nature of the experiments that continued for the expected lifetime of the mice; however, we could not determine bone, MAT and HBM volumes at the beginning. We used a recently validated non-invasive method to quantify MAT *in situ* and analyzed a large number of CTs (~100 slices per femur) to cover both trabecular and cortical zones. Albeit, MAT variations may affect the measurements of bone density in single energy CT [39], but visual checking of the images were confirmatory of the reliability of the image analysis results. We did not measure the degree of fat infiltration into bone compartments (trabecular vs cortical), but based on our image analyses (Figure 3), both components, as well as HBM are impacted by fat infiltration. Future studies will greatly benefit from inclusion of other regions of interest (e.g. vertebral bodies), that would assist in clarifying which regions and component(s) of bone (trabecular or cortical) is affected most by MAT infiltration.

In conclusion, this study provides further evidence on the possible roles of MAT expansion in the progression of age-related bone loss and anemia via the lipotoxic effects of MAT on bone and HBM in aging mice. Further molecular studies are required to investigate the crosstalk between tissues and the mechanisms that MAT affects both tissues.

## MATERIALS AND METHODS

### Ethics

All animal procedures were approved by an animal welfare committee and were conducted in accordance with the National Research Council's guidelines for the care and use of laboratory animals. Animals were housed in the animal laboratory of the INRA Research Center for Human Nutrition. They were housed in a controlled environment (12:12 h light/dark cycle, 20–22 °C, 50–60% relative humidity, 5 mice per cage with free access to water). The weaned puppies were delivered 1 month before study for acclimatization and were provided with free access to a standard growth diet for a month. At the end of the study the mice were euthanized by IP injection of lethal dose of sodium pentobarbital (0.1 ml/gr BW; CEVA Santé Animale, Libourne, France); and tissues were harvested, frozen and stocked prior to investigation.

### Animals

One-month-old female senescence accelerated mouse-prone 8 (SAMP8) were obtained from INRA-Dijon (UMR INRA-CNRS-Université de Bourgogne - Centre des Sciences du Goût et de l’Alimentation (CSGA) Dijon, France). The animals were housed in standard plastic cages as per ethical committee guidelines. After acclimatization, mice were provided with free access to a standard growth diet for a month. Animals were randomly divided in different groups and assigned to different diets for ten months ad libitum. Diet enrichment did not modify daily food intake significantly. To avoid a dramatic increase in SAMP8 mortality (usually at 12 months) of age [40], the duration of study was limited to ten months. Twenty femora and their μCT scans were available per group. Only intact bones were imaged and only images without artefacts were included in the study. Consequently, n=10, 20, 11 and 12 samples were analyzed for control, FO, SFO and borage oil groups, respectively.

### Diets

Diets were purchased from INRA (Jouy-en-Josas, France) or Harlan (Ganat, France). All diets were adjusted to be approximately isocaloric (Δb5%). The mice were fed either a standard growth diet (Harlan Teklad Global 2019), or a sunflower oil (SFO) enriched diet that modulates the fatty acid composition in favour of ω6 (Table 3). Borage oil and fish oil were used to test the effect of a γ-linolenic acid (18:3 ω6; GLA) enrichment or to reduce the ω6/ω3 ratio by providing eicosapentaenoic acid (20:5 ω3; EPA) and docosahexaenoic (22:6 ω3; DHA) respectively.

**Table 3:** Formulations of the diets given to for study groups.

**Ingredient (g/100 g diet)**	**Standard diet (Harlan 2019)**	**Sunflower oil based diet**	**Borage oil enriched diet**	**Fish oil enriched diet**
Wheat starch	55.24	59.14	56.79	56.79
Casein	19.00	19.20	19.20	19.20
Sucrose	4.16	4.16	4.16	4.16
Fiber (cellulose)	3.60	3.50	3.50	3.50
DL methionine	3.30	0.30	0.30	0.30
Choline bitartrate	0.20	0.20	0.20	0.20
Mineral mix	3.50	2.50	2.50	2.50
Vitamin mix	1.00	1.00	1.00	1.00
Sunflower oil	0.00	5.45	5.62	6.73
Canola oil	0.00	2.73	3.37	3.37
Borage oil	0.00	0.00	2.79	0.00
Fish oil	0.00	0.00	0.00	1.68
Oleisol oil	0.00	1.82	0.57	0.57
Soybean oil	10.00	0.00	0.00	0.00
Energy (cal)	420	428	440	440

From I.N.R.A. (Jouy en Josas, France); casein (Union des caséinerie, Surgères, France), cornstarch (Cerestar, Saint-Maur, France), cellulose (Durieux, Marne la Vallée, France), oil (Bailly, Aulnay sous Bois, France), vitamin mixture (Roche, Neuilly sur Seine, France), mineral mixture (Prolabo, Fontenay sous bois, France), DL-methionine and choline bitartrate (Jerafrance, Jeufosse, France).

### Imaging and tissue volume quantification

Frozen femora were stripped of soft tissues and scanned using eXplore CT 120 μCT scanner (GE Healthcare, Canada). Acquisition consisted of 360 views acquired in 1° increments collected in one full gantry rotation, with 20 ms exposure/view. X-ray tube was set at 100 kV and 50 mA. CT images were reconstructed using a modified cone-beam algorithm with an isotropic voxel of 45 μm. One hundred μCT slices distal femora were analyzed per sample; and bone volume fraction, HBM% and MAT% were quantified as follows (Figure 1).

μCT images were saved in DICOM format. All CT slices within the region of interest were analysed by a single blinded observer using Slice-O-Matic^®^ (Tomovision, Montreal, Canada), previously validated for the measurement of various tissue volumes in different species [1, 41–43]. CT Hounsfield values for bone, MAT and HBM tissues were calculated. We used protocols previously validated in animals to quantify yellow/red marrow and bone using a single energy CT (MicroCT) and image analysis using Slice-O-Matic software [43]. Previous publications on the tissue thresholds were consulted, and final threshold ranges used for quantification of these parameters were further refined visually, using an image histogram [43–47]. Thus, the following resultant CT number thresholds were applied: MAT ≤450; HBM 451-1900; and bone ≥1901 Hounsfield units (Figure 3).

Ratios of tissue volumes/total tissue volume (i.e. bone%, HBM% and MAT%) and ratios of HBM/total marrow% and MAT/total marrow% volumes were calculated.

### Statistics

Data are presented as median and interquartile range. Scatter plots were visually inspected to confirm linear relationship between variables and Pearson’s correlation coefficient is reported. Between group comparisons were performed using Kruskal-Wallis test with post hoc rank sum tests for pairwise comparisons with p values adjusted using Bonferroni procedure. Analyses were performed using Stata 15.1 (StataCorp. 2017. Stata Statistical Software: Release 15. College Station, TX: StataCorp LLC). Significance was set to *p*≤0.05; and for multiple comparisons *p* was set to ≤0.001.
